# Suggestions with Reference to Cases of Incurable Lunacy

**Published:** 1853-04-01

**Authors:** 


### SUGGESTIONS WITH REFERENCE TO CASES OF INCURABLE LUNACY.

(*From Appendix to Report of Commissioners on the Law of Divorce.*)

The Commissioners may not be unnecessarily enlarging the scope of their very important inquiries in considering the cases of married persons, in which either the husband or wife has sunk into a state of hopeless lunacy; nor may this question be unworthy of their regard, whether contemplated from a moral and religious, or even merely from a physical point of view.

The force of the marriage contract is granted; all that is bound up in this relation is admitted.

The only question is, as to the degree of sanction derivable from the force of law, divine and human, for isolating for life the husband or wife of a lunatic in cases of *confirmed* and *incurable* lunacy.

To all intents and purposes such lunatic is dead in law. It is a legal wrong, so to speak, and a positive moral and physical deprivation to the party legally connected with one thus dead in the eye of the law, to be compelled to hold to that person through life.

The consequences and consequent practices are, from natural causes, evil in their nature and effect as regards the wife or husband of the lunatic, without any good result to the lunatic.

It is, of course, impossible to enter into details; but the course of life led by the husbands or wives of lunatic persons, in known instances, renders the presumption strong that in the great majority of cases Nature asserts and vindicates her claims.

Take the case of a sane man, in the prime of life, full of health and vigour, chained to such forced and unnatural celibacy—take the case of a young sane female similarly circumstanced — in both examples physical requirements receive an unwonted impulse of intense power; and thus, from the very circumstances of the case, the marriage, although in fact null, is productive of some of the worst evils to society.

In relieving the greatest sufferers — those, namely, whose suffering is rendered more acute by the consciousness that it is imposed upon them by the law—should we not at the same time, by harmonizing law with the abstract principles of justice, while benefitting society, be upholding the majesty of law itself?

Is it not cruel, then, to impose upon either the husband or wife of a lunatic, whose physical and mental powers are gone for ever, a celibate state, unnatural and unnecessary, and too frequently immoral in its results?

Such arrangements might readily be made as would secure to a *lunatic's children* any property to which they might be entitled, and due provision might be made for *the maintenance* of the *lunatics themselves.*

A release or divorce permitting a second marriage, even though the lunatic be alive, might then be deemed, and be legal, without prejudice to the incurable lunatic person and his or her children.

All which those who advocate a change in the law in this respect can desire, is, that when instances occur which require judicial investigation, the tribunal resorted to should be competent to conduct that investigation, and to grant the best remedy which in such cases can be afforded. It should be the duty of those presiding in such a tribunal never to allow the dissolution of the nuptial contract unless it clearly appeared that its continued maintenance would be injurious, not only to the parties themselves, but to society; and it is submitted, therefore, that the Commissioners, weighing the *interests of lunatic persons*, and of their non-lunatic husbands or wives, and the welfare also of their children, should, if possible, suggest to the Crown and legislature such a remedy as will meet the exigencies of these melancholy cases.

